# Unidirectional Moisture Delivery via a Janus Photothermal Interface for Indoor Dehumidification: A Smart Roof

**DOI:** 10.1002/advs.202301421

**Published:** 2023-05-17

**Authors:** Wenbo Shi, Haoyu Bai, Moyuan Cao, Xinsheng Wang, Yuzhen Ning, Zhe Li, Kesong Liu, Lei Jiang

**Affiliations:** ^1^ School of Materials Science and Engineering Smart Sensing Interdisciplinary Science Center Nankai University Tianjin 300350 P.R. China; ^2^ School of Chemical Engineering and Technology Tianjin University Tianjin 300072 P.R. China; ^3^ Haihe Laboratory of Sustainable Chemical Transformations Tianjin 300072 P. R. China; ^4^ School of Chemistry Beihang University Beijing 100083 P.R. China; ^5^ Tianmushan Laboratory Hangzhou 310023 P.R. China

**Keywords:** unidirectional wettability, Janus interfaces, fluid manipulations, solar evaporations, solar dehumidification

## Abstract

Rational control of the humidity in specific environments plays an important role in green building, equipment protection, etc. A smart apparatus that can actively expel inner moisture and largely prevent outer liquid penetration can be highly desirable. Through the integration of the Janus interface with unidirectional liquid manipulation and the solar evaporating layer, here, a Janus solar dehumidifying interface (JSDI) is designed for the switchable moisture management of an indoor environment. By covering with the JSDI roof, the continuous elimination of inner water is achieved via outward condensate delivery and solar evaporation on sunny days. On rainy days, JSDI with a hydrophobic lower surface can largely hamper inward liquid leakage and then spontaneously drain the accumulated water via a siphoning structure. The real‐world water evaporation rate via the JSDI is ≈0.38 kg m^−2^ h^−1^ on an autumn day, showing a promising function of in situ moisture expelling. In addition, the JSDI is made of natural materials that are easy to scale up with a cost of four dollars per square meter. It is envisioned that the JSDI may meet the wide requirements of indoor dehumidification and update the understanding of the integration of Janus interfaces and solar steam generation.

## Graphical abstract

Through the integration of the unidirectional liquid penetration of Janus interface and the interfacial solar evaporation, here a Janus solar dehumidifying interface is presented. The complex structure enables a switchable function between sunny and rainy days, which can be utilized as a smart roof for moisture management.

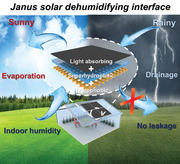

## 1 Introduction

The development of modern science and technology requires a more efficient approach to controlling the parameter of environment niches such as temperature, humidity, chemical concentration, etc. As a possible solution, a fluid‐manipulating interface is applicable to control the distribution, injection, and separation of liquid and gas, which offers us great opportunity to improve the systems relating to multiphase interactions^[^ ^1^ ^]^ With an appropriate strategy, both the efficiency and the reliability of functional systems can be facilely optimized via the incorporation of such interfaces^[^ ^2^ ^]^ For example, the continuous gas supply through a superaerophilic mesh is able to largely increase the accuracy of enzyme sensors^[^ ^3^ ^]^ and the efficiency of TiO_2_‐based photocatalysis^[^ ^4^ ^]^ originating from the enhanced and sustainable oxygen concentration around the active site. The microscale interface with wettability contrast can boost the mass transfer around the catalyst, and then influence the selectivity and the efficiency of chemical conversion in the field of CO_2_ electroreduction^[^ ^5^ ^]^ Fischer‐Tropsch synthesis^[^ ^6^ ^]^ water splitting,^[^ ^7^ ^]^ etc. With respect to open‐surface microfluidics, the liquid controlling system can promote high‐performance liquid/vapor exchange and high‐throughput analysis, which should unlock more options to construct superior platforms for real‐world applications such as microreactors^[^ ^8^ ^]^ liquid delivery^[^ ^9^ ^]^ heat transfer,^[^ ^10^ ^]^ etc.

To facilitate the on‐surface fluid transporting process without energy input, three types of bioinspiration can be taken into consideration, i.e., the Janus wettability of lotus leaf^[^ ^11^ ^]^ the oriented micro/nanostructure of lizard skin^[^ ^12^ ^]^ and the geometric gradient of cactus spine^[^ ^13^ ^]^ From the mechanism of driving factors, the asymmetric Laplace pressure and the directional release of surface energy can be attributed to the propulsion of the fluid self‐transport^[^ ^14^ ^]^ Among the fluid self‐transporting processes, the unidirectional fluid penetration on the Janus porous interface has drawn wide attention due to its promising application as fluidic diode^[^ ^15^ ^]^ anti‐gravity liquid delivery^[^ ^16^ ^]^ selective fluid gating,^[^ ^17^ ^]^ etc. By tuning the interfacial porosity and thickness, fluids can actively pass through the Janus interface from the lyophobic side to the lyophilic side, which is driven by the gradient of surface energy. In the opposite direction, fluids tend to spread on the lyophilic surface instead of penetration. Taking advantage of this manipulating logic, versatile fluids such as liquid‐in‐air,^[^ ^18^ ^]^ oil‐in‐water,^[^ ^19^ ^]^ and gas‐in‐water^[^ ^20^ ^]^ can fulfill a one‐way transport on the Janus interfaces with varying structures. After unraveling the mystery of this unidirectional liquid penetration, one of the most important topics is how to extend this fluid‐manipulating interface to an indispensable field in a reasonable way.

Within the past decade, interfacial solar evaporation has been widely investigated due to its bright future in seawater desalination^[^ ^21^ ^]^ atmospheric water harvesting,^[^ ^22^ ^]^ etc. Through the light localization strategy, the evaporating process can be significantly accelerated at air/water interface. The incorporation of fluid‐manipulating surfaces into solar evaporation systems can remarkably optimize their performance, originating from the effective separation of evaporating surface and water source. This water pumping process is fundamental to the lowering of heat dissipation and the localization of solar irradiation^[^ ^23^ ^]^ In addition, the Janus wettability and the asymmetric structure can endow the evaporator with salt‐rejecting ability^[^ ^24^ ^]^ and interfacial floatability^[^ ^25^ ^]^ indicating the high adaptiveness of the Janus feature in the design of solar evaporators. The combination of a fluid‐manipulating interface with photothermal materials should open a new avenue to innovate advanced devices and diversify the application strategy.

**Figure 1:**
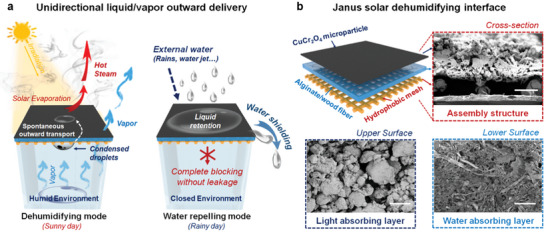
The unique indoor dehumidifying function of JSDI. a) The unidirectional liquid/vapor delivery process in dehumidifying mode and water repelling mode. On sunny days, with the heating effect of the light‐absorbing layer, the evaporated vapor can freely pass through the JSDI, resulting in a decrease in indoor humidity. On rainy days, raindrops cannot penetrate the JSDI from outside but are drained instead, preventing the room from getting wet. b) The design and fabrication of a typical JSDI are composed of three layers, including the light‐absorbing layer of CuCr_2_O_4_ microparticles, alginate‐based superhydrophilic layer, and hydrophobic copper mesh. The SEM images show the morphologies of the JSDI. The scale bar of the assembly structure is 500 µm, and that of the light‐absorbing layer and the water‐absorbing layer is 200 µm.

The indoor dehumidifying process commonly relies on an air conditioner or moisture absorbent, which requires external energy to power up and regenerate active ingredients. If an apparatus can achieve continuous moisture removal without any energy input or consumables, the dehumidifying process should be simplified and economical, which can also meet the requirement of carbon neutrality. Through the integration of unidirectional liquid penetrating logic and interfacial solar evaporation, here we report a multilayered Janus photothermal interface for sustainable indoor dehumidifying. The proposed Janus solar dehumidifying interface (JSDI) can continuously and spontaneously drive the anti‐gravity delivery of condensed droplets, facilitating a unidirectional outward fluid transport. On the basis of the superhydrophilic/hydrophobic cooperative structure, the indoor vapor can freely pass through the JSDI, whereas the external liquid cannot penetrate the JSDI from outside. With the assistance of a light‐absorbing layer on the upper surface of JSDI, the transferred liquid can be rapidly vaporized on sunny days, resulting in a continuous process of indoor dehumidification (**Figure** **1**a). Even under liquid dripping or jetting, the JSDI can prevent inward liquid penetration and guide water shielding, showing an available water‐repelling process on rainy days. The current design possesses a fixed structure and a switchable function, which is suitable for integration with advanced systems as an energy‐independent dehumidifying apparatus. We envision that the incorporation of unidirectional liquid manipulation should extend the applications of interfacial solar evaporation, and stimulate new thinking in the field of moisture management, water harvesters, green buildings, etc.

## 2 Result and Discussion

### 2.1 Design and Fabrication of JSDI

Considering the cost performance and the practical applications of JSDI, we have selected abundant natural materials with lost cost and flexibility. Furthermore, the fabricating process should not involve any toxic reagents or solvents, which are scalable, facile, and environmentally friendly. As shown in Figure 1b, the typical JSDI is composed of three layers including the light‐absorbing layer of CuCr_2_O_4_ microparticles, alginate‐based superhydrophilic layer, and the hydrophobic copper mesh. The inorganic dye, copper chromite black, is chosen as the light‐absorbing component due to its high stability and efficient solar‐to‐heat conversion. For the construction of the lower surface, the copper mesh with large pores was first modified with polydimethylsiloxane (PDMS) to enhance the hydrophobicity. Subsequently, the hydrophilic slurry composed of sodium alginate and wood fiber was uniformly deposited on the upper surface of the hydrophobic copper mesh. To strengthen the interfacial adhesion, the upper surface of hydrophobic copper mesh was abrased prior to the painting process. After coating the hydrophilic slurry, a certain amount of CuCr_2_O_4_ particle with an average size <2.5 µm was directly sprinkled on the hydrophilic slurry with a sieve, resulting in an areal density of 250 g m^−2^. Finally, the dried multilayered interface was crosslinked in CaCl_2_ solution and then washed to remove untethered particles. The three‐layered assembled structure can be clearly verified by scanning electronic microscopy (SEM). The aggregate of CuCr_2_O_4_ particles automatically assembles into capillary structure for liquid pumping. The alginate‐based polymer was blended with the wood fiber with a diameter of 40 µm, forming the water‐absorbing layer. The as‐prepared hydrophilic substrate has a relatively dense structure that can provide sufficient mechanical strength. The hydrophobic copper mesh was coated with a thin layer of silicone, exhibiting a smooth surface (Figure S1, Supporting Information). The contact angle of hydrophobic copper mesh is 140.4° ± 1.5°, which can facilitate liquid support and thorough droplet dewetting. The interfacial adhesion between the hydrophilic layer and the hydrophobic mesh was between 7–9 N (Figure S2, Supporting Information), and CuCr_2_O_4_ particle was more stably adhered to the deposited layer with alginate and wood fiber (Figure S3, Supporting Information).

The hydrophobic porous substrate can prevent water penetration due to the upward Laplace pressure from the curve surface of droplet. The pore size and the surface hydrophobicity contribute to the critical height of liquid support, which can be calculated by the following Equation (1):

(1) $$P_{intrusion}=\frac{4\gamma_{water}\cdot \left|cos\theta_{ca}\right|}{D_{pore}}$$

where 𝛾_water_ is the surface tension of water, *D*_pore_ is the pore diameter of the mesh, and *θ*_ca_ is the water contact angle of the hydrophobic mesh. In this work, the PDMS‐coated copper mesh has an intrinsic contact angle exceeding 140°, and three kinds of copper meshes with maximum pore sizes of 195, 257, and 392 µm were carefully tested (Figure S4, Supporting Information). All of the hydrophobic meshes can support a liquid column exceeding 10 mm which is enough for the liquid shielding process (*P*_intrusion_ *= h*_max_ *× ρ*_water_ *× g*). Therefore, the large‐pore copper mesh was selected in the following experiment due to the high accessibility of condensed droplets. Alginate and wood fiber are common natural products, and the CuCr_2_O_4_ powder is one of the most successful inorganic black dyes with high thermal stability. Building on the rational design of multilayered structure, the as‐prepared JSDI can achieve a continuous process of vapor condensation, anti‐gravity droplet delivery, and interfacial solar evaporation.

### 2.2 Unidirectional Liquid Manipulation of JSDI

**Figure 2:**
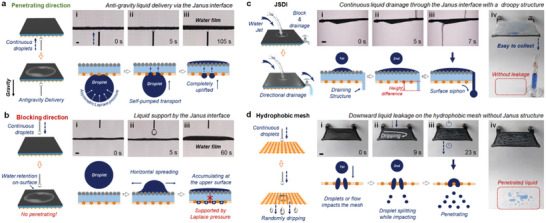
Unidirectional liquid manipulation of JSDI. The continuous flow with a flux of 20 mL min^−1^ was incorporated from a) the lower and b) the upper surfaces of JSDI by a hydrophobic needle. The liquid will penetrate directly from the hydrophobic surface of JSDI to the hydrophilic surface under the assistance of asymmetric Laplace pressure, regardless of gravity. However, in the opposite direction, the droplet can only spread on the horizontal surface, and the hydrophobic pores effectively support the accumulated liquid. The scale bar is 1 mm. c) Even under a dynamic water flow, the downward liquid penetration is still blocked by JSDI. The accumulated water is drained and collected via a hydrophilic siphoning structure. The scale bar is 1 cm. d) In contrast, without the buffering of the superhydrophilic layer, the droplets which were released from >1 cm high will partially pass through the hydrophobic mesh upon impact, resulting in the failure of liquid shielding. The scale bar is 1 cm.

Asymmetric interfaces with Janus wettability provide a useful method to realize unidirectional liquid penetration. For JSDI, the complex structure of hydrophobic copper mesh and the hydrophilic water‐absorbing layer is a typical Janus interface with large pores. Liquid can easily penetrate the JSDI from the hydrophobic mesh to the hydrophilic layer (penetrating direction), whereas the penetrating process failed in the opposite direction (blocking direction). This unidirectional liquid penetration is determined by the gradient of surface energy, which can be simulated using COMSOL Multiphysics (Figure S5, Supporting Information). In our previous report, we proved that the superhydrophobic mesh with large pores (≈0.5 mm in diameter) is able to assist a continuous self‐pumping of droplets^[^ ^16a^ ^]^ The similar design can be applied to the outward droplet transport during the vapor condensation. To test the anti‐gravity delivery of JSDI, water droplets were continuously charged to the lower surface of JSDI (**Figure** **2**a; Movie S1, Supporting Information). Droplets are dragged by the hydrophilic layer through the large pores of hydrophobic mesh, completing a unidirectional anti‐gravity delivery. As a result of continuous liquid pumping, the uplifted water starts to accumulate on the upper surface of JSDI. From the view of mechanism, the hanging droplet with a spherical shape possesses considerable surface energy. The thickness of the hydrophilic layer does not affect the experimental effect, and the anti‐gravity transport can be achieved for different thicknesses (1, 2, and 3 mm) of the hydrophilic layer. The thickness of the hydrophobic copper mesh also does not affect the experimental effect, but the pore size of the copper mesh is too small to cause the droplets to be blocked, so a copper mesh with a larger pore size of 20‐mesh was chosen to design the experiments in this experiment (Figure S6 and Movie S2, Supporting Information). Once the droplet contacts the hydrophilic layer, the asymmetric Laplace pressure from the curve surface of droplet will propel the upward mass transfer. In the opposite direction, while droplets were discharging from the top of JSDI, water tends to spread horizontally and accumulate at the upper surface without any downward leakage (Figure 2b; Movie S3, Supporting Information). The hydrophobic pores of lower surface are capable of hindering the liquid penetration because of the upward Laplace pressure.

The desired JSDI should not only block the reversed penetration but also actively drain the accumulated water to the desired site. To guide the water drainage, a hydrophilic surface siphoning structure of liquid drainage was easily added to the JSDI (Figure 2c and Movie S4, Supporting Information). Driven by gravity, water residue on the upper surface will be rapidly drained to a specific site through the hydrophilic porous structure, which facilitates a convenient liquid‐collecting process (Figure S7 and Movie S5, Supporting Information). Even under a dynamic water flow, the JSDI can completely repel water from penetrating, because the hydrophilic upper surface can largely suppress the impact of water flow by inducing the horizontal liquid spreading. Noted here, to show the liquid spreading process, we selected the Janus interface without black CuCr_2_O_4_ particle which has the same wettability as JSDI. In comparison, droplets would split into tiny droplets while impacting the mesh without Janus structure, resulting in undesired liquid penetration (Figure 2d; Movie S6, Supporting Information). The reliable unidirectional liquid manipulating ability and the spontaneous water‐draining process should play an important role in the subsequent dehumidifying process.

**Figure 3:**
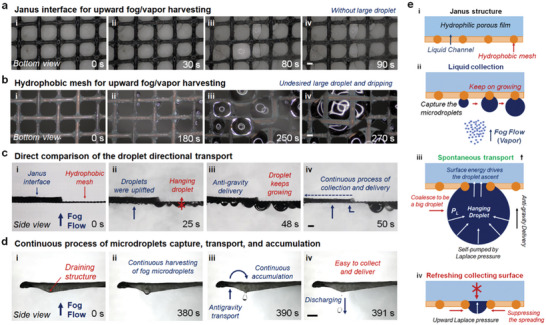
Unidirectional liquid manipulation of the JSDI under fog/vapor collection. a) The fog‐collecting experiment was used to illustrate the availability of the continuous process. In terms of the Janus structure, the fog was first captured by the lower surface of the hydrophobic mesh. The tiny droplets coalesced to form larger droplets, which were subsequently absorbed by the hydrophilic layer. The scale bar is 100 µm. b) In comparison, for the single‐layer hydrophobic mesh, the growing droplets cannot be uplifted but grow larger, and eventually drip downward. The scale bar is 100 µm. c) Direct comparison between the Janus structure and hydrophobic mesh during fog collection. Both interfaces were placed horizontally, and the upward fog flow was applied to the lower surface. In the process of continuous fog collection, the lower surface of the Janus interface remains clear, whereas the lower surface of sole hydrophobic mesh captures many droplets. The hanging droplets located at the boundary of two interfaces can be absorbed by the hydrophilic layer, showing the outstanding water collection ability of the Janus structure. The scale bar is 1 mm. d) The integrated fog collector consists of a Janus structure and a siphoning structure, which can realize the continuous process of fog capture, liquid transport, and water drainage. The scale bar is 1 cm. e) Schematic diagram of the unidirectional fog collection mechanism. Tiny droplets of the mist are captured by the hydrophobic mesh and keep on growing. Once the grown droplet contacts the upper hydrophilic surface through the large hydrophobic pore, it will be rapidly uplifted driven by the asymmetric Laplace pressure. Meanwhile, the lower surface was completely refreshed for manipulating the next droplet.

After the investigation of unidirectional liquid control of JSDI, the complicated process of outward moisture transport including microdroplet capture, droplet growth, and antigravity delivery should be taken into consideration. To test the adaptive performance of the JSDI, we conducted a fog‐collecting experiment to show the availability of the continuous process. Under an upward fog flow, microdroplets were first captured by the lower surface of hydrophobic mesh (**Figure** **3**a; Movie S7, Supporting Information). The captured droplet would keep on growing by coalescing with other droplets and hanging on the copper mesh. Once the droplet contacts the upper hydrophilic layer, it will be rapidly uplifted and absorbed. Thanks to the hydrophobic surface of copper mesh, the complete dewetting process during the droplet ascent can avoid the wetting of mesh and then guarantee the continuity of droplet transport (Figure S8 and Movie S8, Supporting Information). The principle of droplets transfer from the juxtaposed single hydrophobic through‐net to the hydrophilic side of the hydrophobic Janus structure is as follows. Tiny droplets of the mist are captured by the hydrophobic mesh and keep on growing. Once the grown droplet contacts the upper hydrophilic surface through the large hydrophobic pore, it will be rapidly uplifted driven by the asymmetric Laplace pressure. Meanwhile, the lower surface was completely refreshed for manipulating the next droplet. This unidirectional droplet transport is durable and not prone to failure. Through experimental observation, the collection of tiny droplets that are unable to converge is hard to achieve. The captured tiny droplets on the copper mesh are not large enough to contact the hydrophilic layer. Upon placement within the fog environment, nearly all the microdroplets are able to converge to form large droplets and be absorbed. When the fog dissipates the tiny droplets will subsequently evaporate and disappear in the environment. The detailed mechanism of the unidirectional microdroplet manipulation was illustrated in Figure 3e, revealing the driving factor of this continuous and spontaneous process. In contrast, the hydrophobic copper mesh without a hydrophilic layer can only realize the microdroplets capturing process (Figure 3b; Movie S9, Supporting Information). Due to the lack of anti‐gravity delivery, the hanging droplet on the hydrophobic mesh is much bigger than that on the Janus structure, which would eventually drip downward.

The different droplet pumping during fog collection can be clearly visualized from a side view (Figure 3c; Movie S10, Supporting Information). The hydrophobic copper mesh was partially covered by the hydrophilic layer and placed in the fog flow. Nearly no visible droplet can be seen under the Janus interface, whereas the droplet tends to hang under the sole hydrophobic mesh. Even if the hanging droplet is located at the boundary of Janus interface, it will be uplifted and transferred from the copper mesh to the upper surface of JSDI structure. The surface siphon process was also effective for microdroplet manipulation, i.e., a complete process including microdroplet capture, droplet self‐pumping, and liquid drainage can be orderly carried out in an orderly manner by one integrated JSDI (Figure 3d; Movie S11, Supporting Information). In addition, we have tested the availability of PDMS coating under hot steam to exhibit the rationality of our design. Results showed that the PDMS coating is able to maintain hydrophobic under 75 °C steam, indicating the stability and sustainability of the designed hydrophobic copper mesh in steam condensation (Figure S9, Supporting Information). Therefore, this Janus interface with liquid manipulating ability was further integrated into a JSDI system for practical application.

### 2.3 The Dehumidifying Performance of JSDI

**Figure 4:**
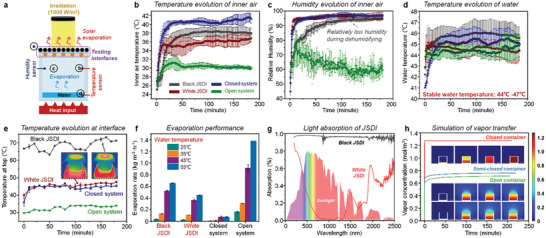
The in‐lab dehumidifying performance of JSDI. a) The dehumidification experimental setup with temperature and humidity sensors. Comparison of the dehumidification performance of the system corresponding to the four kinds of test interfaces (Black JSDI, White JSDI, closed system, open system) as the inner water temperature was fixed at 45 °C. b) The inner air temperature, c) the relative humidity, d) the water temperature, and e) the surface temperature are continuously recorded during the dehumidifying process. (f) The water evaporation rate of different types of interfaces under various inner water temperatures during the 3‐hour test. (g) Ultraviolet–visible‐near‐infrared (UV–vis–NIR) spectra of JSDI between 200–2500 nm. The light absorption of black JSDI and white JSDI are shown. h) COMSOL Multiphysics simulation of the vapor discharge behavior of the closed, semi‐closed (JSDI), and open systems.

Antigravity liquid delivery is quite suitable for the transport of condensed water because of the direction in accordance between vapor and JSDI placement. We first tested the dehumidifying ability of JSDI in laboratory. The dehumidification experimental setup is shown in **Figure** **4**a, where the test interfaces (11 cm × 11 cm) are tightly stacked on a 1 L glass cubic beaker containing 50 mL water (Figure S10, Supporting Information). For mimicking the actual environment, a heating plate is placed under the glass beaker to control the water temperature in the container. Simulated solar light with a density of 1000 W m^−2^ is irradiated on the testing interfaces to accelerate the interfacial evaporation. Three sensors were placed inside the container, including two temperature sensors inside the container for recording air temperature and water temperature respectively, and one humidity sensor to evaluate the variation in moisture of inner air. Four kinds of testing interfaces were selected as the JSDI with CuCr_2_O_4_ layer (Black JSDI), JSDI without CuCr_2_O_4_ layer (White JSDI), glass plate (closed system), and control group (open system). The inner water temperature was tuned to range from 25 °C to 55 °C to simulate the different environments corresponding to different seasons. Each dehumidifying experiment was conducted for 3 h and the data were recorded every minute.

The inner air temperature of different systems is continuously recorded during the dehumidifying process. As the inner water temperature is controlled at 45 °C, the closed system has the highest temperature of ≈40 °C, whereas the temperature of the open system is ≈30°C (Figure 4b). Compared with the closed system, the incorporation of black JSDI and white JSDI can reduce the inner air temperature by 2–5 °C due to the shading of solar irradiation. Black JSDI generates a higher inner air temperature (≈3 °C) than white JSDI owing to the higher surface temperature (Figure 4e; Figure S11, Supporting Information). The thermal conductivity of the hydrophilic layer is 0.165 ± 0.004 W m^−1^ K^−1^, similar to pine (0.17 W m^−1^ K^−1^) and cardboard (0.14 W m^−1^ K^−1^). In the whole process of dehumidification, the inner water temperature remains at 44–47°C (Figure 4d), indicating the higher evaporation is mainly dependent on the acceleration of vapor discharge. As shown in Figure 4c, the relative humidity of the white JSDI and the closed systems increased rapidly to 90% within a short period of time (10 min), and the humidity of black JSDI system has a relatively low increasing rate. The black JSDI has a higher surface temperature should accelerate the vapor transfer, resulting in a show humidity increase. However, after one hour experiment, the humidity of above systems exceeds 90% due to the massive evaporation of bottom water, and only can a slight difference be found between white and black JSDIs. In comparison, the open system that was directly connected to the open air had a low humidity of 60%.

The performance of dehumidification can be quantitively evaluated by the water loss in the container (Figure 4f). After 3 h of experiment, the mass change of water was recorded under varied inner water temperatures of 25, 35, 45, and 55 °C (Figure S12, Supporting Information). The initial volume of water is set as 50 mL, and water temperature is controlled by the heat plate. The closed system shows the lowest mass of water evaporation (≈0.1 kg m^−2^ h^−1^), indicating the vapor cannot effectively escape from the container. As we expected, the open system has the fastest evaporation under all temperatures. At a water temperature of ≈55 °C, the water was just evaporated completely for open systems. Interestingly, both black JSDI and white JSDI achieve effective dehumidification during the experiment. The water loss of black JSDI was more than that of white JSDI, and this difference is gradually magnified as the water temperature is increasing from 25 °C to 55 °C. At a low water temperature of 25 °C, the water loss rate of black and white JSDIs is 0.04 kg m^−2^ h^−1^ and 0.03 kg m^−2^ h^−1^ respectively. As the environment temperature increased to 35 °C, the water loss of black and white JSDIs was enhanced to 0.13 kg m^−2^ h^−1^ and 0.11 kg m^−2^ h^−1^. The higher water temperature promotes a higher efficiency of interfacial dehumidification. At the water temperature of ≈45 °C, the efficiency of black JSDI (0.52 kg m^−2^ h^−1^) was 1.39 times that of white JSDI (0.37 kg m^−2^ h^−1^). The biggest difference can be found at 55 °C, at which the efficiency of black JSDI (0.65 kg m^−2^ h^−1^) was 1.46 times that of white JSDI (0.45 kg m^−2^ h^−1^). The actual interfacial temperature of black JSDI remains at 70 °C, and the white JSDI shows no obvious difference from the closed system (≈45 °C). The enhanced interfacial temperature is attributed to the high absorption of solar irradiation of CuCr_2_O_4_ layer (Figure 4g). We have compared the light‐to‐heat conversion efficiency of black and white JSDIs through an indoor experiment (Figure S13, Supporting Information). The evaporation rate was measured under one sun irradiation, the water mass change was recorded every 30 min. The evaporation rate of black JDSI reached ≈1.51 kg m^−2^ h^−1^ with a conversion efficiency of ≈90.8%. In contrast, the evaporation rate of white JDSI only reaches ≈0.53 kg m^−2^ h^−1^ with a conversion efficiency of ≈27.6%. The conversion efficiency (*η*) was calculated according to Equation (2)^[^ ^26^ ^]^

(2) $$\eta =\frac{m\left(C_{p}\times \Delta T+\Delta H_{vap}\right)}{C_{opt}I}$$

where *m* is the equilibrium water evaporation rate (kg m^−2^ h^−1^). *C_p_* is the specific heat capacity of water (4.18 J g^−1^ K^−1^). Δ*T* represents the temperature difference between vapor and ambient conditions. Δ*H_vap_* is the enthalpy of water evaporation that is 2257 kJ kg^−1^. *C*_opt_ is the concentration of light, and *I* represent solar illumination.

Although the JSDI may hamper the outward transport of inner vapor, the simulation result reveals that this semi‐closed system can also allow the vapor discharge similar to the open system (Figure 4h; Movie S12, Supporting Information). Only a slight difference in the outside vapor concentration (≈0.1 mol m^−3^) between open and semi‐closed systems is displayed. The JSDI can actively and efficiently reduce the moisture indoors on a hot day, which fits well with the condition of the daytime of summer. Besides, this JSDI can also be utilized to reduce the indoor moisture of massive inventory rooms, greenhouses, sunshine rooms, etc.

### 2.4 The Outdoor Experiment of JSDI for the Practical Dehumidifying Process

**Figure 5:**
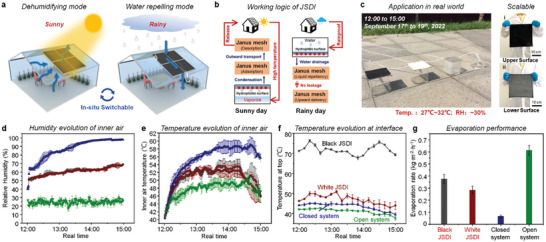
The real‐world dehumidifying performance of JSDI on sunny days. a) Schematic diagram of the smart roof based on JSDI. The dual switchable mode can integrate dehumidification on sunny days and waterproofing on rainy days. b) The dehumidifying and water‐shielding logic of smart roofs in different weather conditions. c) The outdoor experimental setup of the JSDI smart roof. The tested roof is placed on the ground under direct sunlight. The environmental temperature is ≈27–32 °C, and the outdoor relative humidity is approximately 30%. The fabrication of JSDI roof can be scaled up. The scale bar is 10 cm. Comparison of the dehumidification performance of the system corresponding to the four kinds of test interfaces (Black JSDI, White JSDI, closed system, open system). d) The relative humidity, e) inner air temperature, and (f) surface temperature are continuously recorded during the dehumidifying process. g) The water evaporation rate of different containers under real solar irradiation in 3 h.

On the basis of the unique liquid manipulation and solar evaporation, the JSDI can be designed as a smart roof for switchable and energy‐saving dehumidification (**Figure** **5**a). Only with a fixed smart roof, can switchable performance be achieved automatically. On sunny days, the evaporated water indoors can be condensed by the lower surface of JSDI and spontaneously transported to the upper surface, and then the moisture can be discharged, viz. a dehumidifying mode. On rainy days, the Janus structure of the smart roof can efficiently shield the water and guide water drainage with the surface siphon effect, viz. a water‐repelling mode (Figure 5b). The current JSDI is more likely a window for vapor expelling, which can be used to control and lower the room humidity. However, additional investigation is required to ascertain its efficacy in temperature control. The dehumidifying performance of JSDI smart roof has been tested in a real environment on a sunny day (Figure 5c). The JSDI can be easily scaled up to 25 cm × 25 cm with an estimated cost of four dollars per square meter, and the structure of JSDI keeps stable during the processing. The estimated cost of the design is summarized by calculating the purchase price of the raw materials and reagents required to fabricate JSDI. The outdoor experiment was conducted from Sept. 17th to 19th (2022) which is a sunny day with a mild temperature ≈30 °C. We evaluated the dehumidifying performance at the strongest sunlight exposure time from 12:00 to 15:00 each day. The temperature and the humidity were recorded continuously, and the water evaporation rate of different systems has been compared. The inner humidity of closed and semi‐closed systems increases gradually, and the open system seems no change (Figure 5d).

The initial humidity of black JSDI, white JSDI, and closed system are all ≈40%. In a short period of time, the humidity of the closed system increased to ≈65%, and the humidity of the black JSDI and white JSDI increased to ≈50%. After one‐hour solar dehumidification, the humidity of closed system reaches 90%, whereas the JSDI systems can control the humidity ≈60%. This trend of humidity change is slightly different from that of the above test in laboratory. The main reason for this distinction can be attributed to the varied heating strategy of laboratory and real environment tests. In the real environment, the container was largely heated by sun irradiation due to the diffuse reflection. However, in the laboratory, we can only heat the system from the bottom. Therefore, as shown in Figure 5e, the inner air temperature of the real‐world experiment is much higher than that of in‐lab experiment (>10 °C.). The interfacial temperatures of the outdoor experiment are similar to those of the in‐laboratory experiment, indicating that the solar evaporating process is not the main factor of enhanced inner air temperature (Figure 5f). The date of water loss can provide important evidence for the dehumidifying process (Figure 5g). The results of the outdoor experiment are consistent with the in‐lab experiment. The open system undoubtedly realized the highest water loss of 45%, and the closed system only lost 5% water after 3 h. The water evaporation efficiency of black JSDI (≈0.38 kg m^−2^ h^−1^) is 1.3 times that of the white JSDI (≈0.28 kg m^−2^ h^−1^). The water evaporation rate in the real world is ≈0.2 kg m^−2^ h^−1^ lower than in‐lab. The main reason for the difference in evaporation efficiency is the different heating processes. For evaporation experiments in outdoor environmental conditions, the environmental conditions are changing and unstable, and the corresponding temperature and humidity change accordingly. On the contrary, for evaporation experiments in the laboratory, a heating apparatus is employed to stabilize the water temperature in a certain range. Both black and white JSDI can accelerate indoor dehumidification, which offers more options to build the patterned smart roof. As we imagined, if this smart roof of JSDI was assembled on buildings or devices, the switchable and continuous dehumidifying process should help us to save energy and control humidity.

## 3 Conclusion

A moisture removal process without energy input is required to build advanced and economical systems. In this contribution, a solar dehumidifying interface with Janus wettability has been reported to achieve spontaneous and continuous liquid/vapor outward delivery. The unidirectional liquid manipulation of Janus wettability can regulate moisture transport, and the solar evaporating ability on the upper surface of JSDI is able to accelerate vapor generation. Taking advantage of the hydrophobic porous substrate, water cannot penetrate inside the container covered with JSDI, showing a water shielding phenomenon. To demonstrate the dehumidifying effect, this integrated interfacial apparatus was tested both in the laboratory and outdoors. The results showed that the JSDI can effectively eliminate the inner water at a rate up to 0.65 kg m^−2^ h^−1^. This design inspires us to propose a smart roof for moisture removal on sunny days and water repellency on rainy days, which can be automatically switched without any structural or wettability changes. The JDSI can extend the research scope of both the Janus interface and solar evaporation, which should meet the requirement in the field of green buildings, intelligent factories, and equipment protection.

## 4 Experimental Section

### Materials and Characterization

Wood fiber (FB‐300) was purchased from Shanghai Yingjia Industrial Development Co. Ltd. Sodium alginate, hydrochloric acid, sodium hydroxide, ammonium persulfate, CuCr_2_O_4_ microparticles, calcium chloride, and other chemicals were purchased from Shanghai Macklin Chemical Co. Ltd. and used without further treatment. Polydimethylsiloxane (PDMS) Sygard 184 was purchased from Dow Corning Co. Ltd.

The scanning electron microscope (Apreo S LoVac, FEI Co. Ltd., America) was used to observe the surface morphology of the hydrophobic mesh, the CuCr_2_O_4_ microparticles, and the alginate/wood fiber. The syringe pump (SP‐2000, Annol Co.) with a constant flow of 20 mL min^−1^ was used to discharge water droplets. The water contact angles of the surfaces were measured by an SDC‐200 geometer (SFMIT Co., China) with a 4 µL droplet. The detailed droplet‐growing processes were recorded by an industrial microscope (GP‐650S, Gaopin Co.). The solar steam generation measurement was performed via a solar simulator (SOLAR‐500, NBeT, China) with an AM 1.5 optical filter. The light intensity was regulated to 1 sun (1000 W m^−2^) by a solar power meter (TM‐207, TENMARS, China). The solar absorption at 200–2500 nm was measured by a UV‒vis‐NIR spectrophotometer (PE Lambda 750, Perkin Elmer, USA) equipped with a 150 mm integrating sphere. The temperature and humidity of the space inside the container in the experiment and the temperature of the water were measured by a temperature and humidity recorder (RS‐WS‐WIFI‐Y4, JIANDARENKE Co., China). A thermal infrared camera (E6, FLIR, USA) was utilized to capture the thermal images. The fog generator (YC‐D205, Yadu, China) with a constant flow of ≈2.2 m s^−1^ and a water content of 0.4 L h^−1^ was used to investigate the performance of the Janus solar evaporating mesh. The interfacial adhesion of the hydrophilic and hydrophobic layers was analyzed using an electromechanical universal testing machine (MTS SYSTEMS CHINA Co.Ltd., CMT8502). The thermal conductivity of the hydrophilic layer is measured using a thermal conductivity meter (TCI, SETARAM, France).

### Preparation of Janus Solar Evaporating Mesh

To fabricate the hydrophobic mesh, the copper mesh was modified with PDMS. First, PDMS prepolymer containing 10% w/w curing agent was mixed with n‐hexane to obtain a 20 wt.% PDMS solution. Subsequently, the copper mesh was repeatedly rinsed with deionized water and ethanol and polished with fine sandpaper to remove the patina, after which it was soaked in 1 mol L^−1^ hydrochloric acid for 5 min and then rinsed with deionized water and dried. The treated copper mesh was soaked in a mixture of 2.5 mol L^−1^ sodium hydroxide and 0.13 mol L^−1^ ammonium persulfate solution for 5 min. When the surface of the copper sheet turned dark blue, it was rinsed with a sufficient amount of deionized water until the effluent was colorless. After being dried, the alkali‐treated copper mesh was dipped into 20 wt.% PDMS/hexane solution for 10 s and then cured in an oven at 100 °C for 1 h.

To fabricate the hydrophilic layer, wood fibers were mixed with sodium alginate dissolved in water in a mass ratio of 1:5. Then, it was poured into a mold for molding. At the same time, a certain amount of CuCr_2_O_4_ particles (≈250 g m^−2^) was directly sprinkled on the coated surface with a sieve. When it was completely dried, it was immersed in 10 wt.% CaCl_2_ solution for 1 h and then washed to remove untethered particles. Finally, the sample was placed in a natural environment to dry. The hydrophilic layer and hydrophobic copper mesh were assembled by attaching the hydrophilic layer covered with CuCr_2_O_4_ particles on the upper surface of the copper mesh while the PDMS on the mesh was not fully cured, and then allowed it to continue curing together, thus completing the three‐layer structure assembly of a typical JSDI.

## Conflict of Interest

The authors declare no conflict of interest.

## Supporting information

Supporting Information

Supporting Information

Supporting Information

Supporting Information

Supporting Information

Supporting Information

Supporting Information

Supporting Information

Supporting Information

Supporting Information

Supporting Information

Supporting Information

Supporting Information

## Data Availability

The data that support the findings of this study are available from the corresponding author upon reasonable request.
