# Supplementary material for: Unidirectional Moisture Delivery via a Janus Photothermal Interface for Indoor Dehumidification: A Smart Roof
Source: Adv Sci (Weinh). 2023 May 17;10(20):2301421. doi: 10.1002/advs.202301421 (PMC10369248; doi:10.1002/advs.202301421)
Supplement: Supplementary file 1 — Supporting Information [file ADVS-10-2301421-s011.pdf]

ADVANCED SCIENCE
Open Access

# Supporting Information

for *Adv. Sci.*, DOI 10.1002/advs.202301421

Unidirectional Moisture Delivery via a Janus Photothermal Interface for Indoor Dehumidification: A Smart Roof

*Wenbo Shi, Haoyu Bai, Moyuan Cao*, Xinsheng Wang, Yuzhen Ning, Zhe Li, Kesong Liu* and Lei Jiang*

Copyright WILEY-VCH Verlag GmbH & Co. KGaA, 69469 Weinheim, Germany, 2016.

# Supporting Information

**Unidirectional moisture delivery via a Janus photothermal interface for indoor dehumidification: a smart roof**

*Wenbo Shi, Haoyu Bai, Moyuan Cao*, Xinsheng Wang, Yuzhen Ning, Zhe Li, Kesong Liu*, and Lei Jiang.*

**Supplementary Figures:**

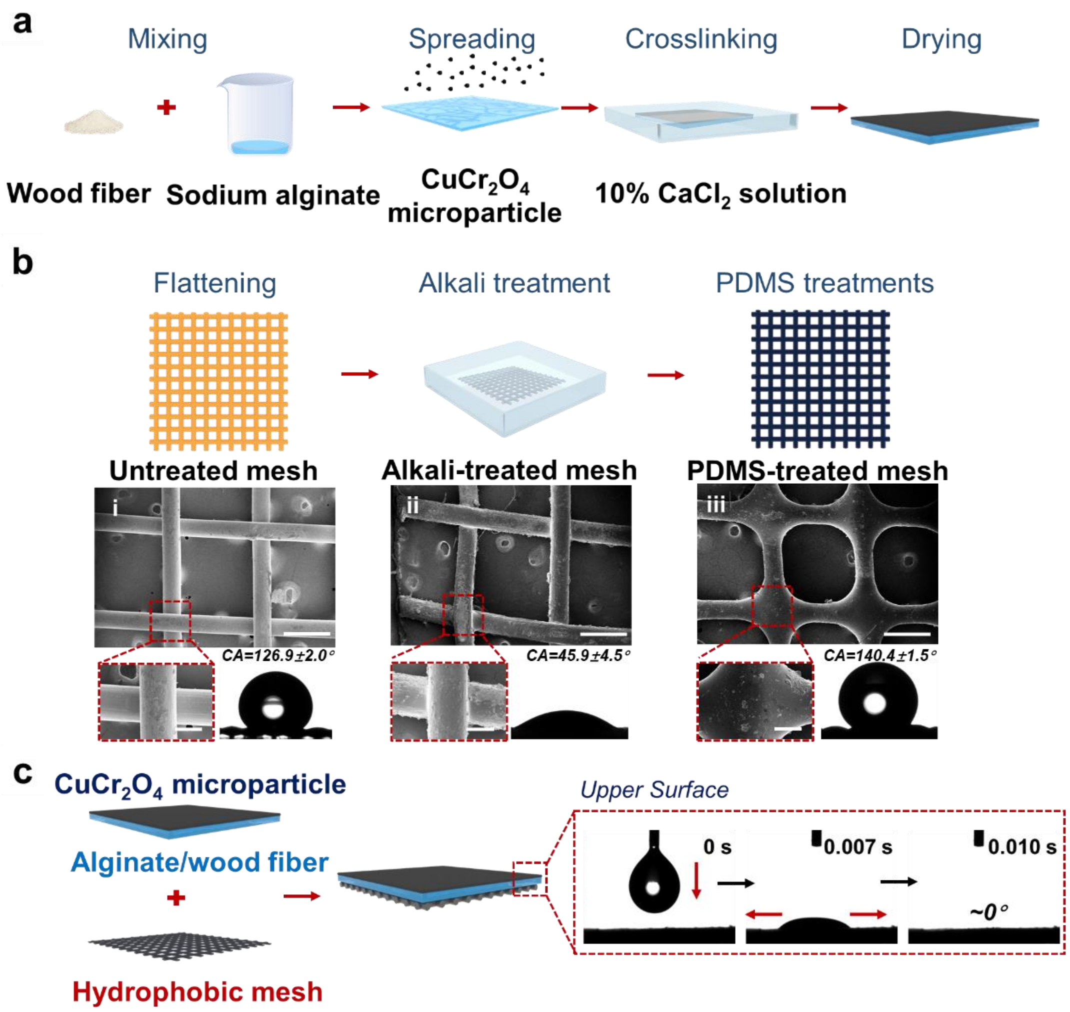


**Figure S1.** The design and the properties of the JSDI. **(a)** The water absorption was prepared by mixing alginate-based polymers with wood fibers, and the light-absorbing layer was prepared from $CuCr_2O_4$ microparticle. The dried multilayered interface was crosslinked in 10% $CaCl_2$ solution and then washed and dried. **(b)** The hydrophobic copper mesh is obtained by subjecting copper mesh (CA=126.9° ± 2.0°) to alkali treatment (CA=45.9° ± 4.5°) followed by PDMS treatment (CA=140.4° ± 1.5°). The SEM images show the different morphologies of the copper mesh. The scale bar is 200 μm, and that of the enlarge image is 50 μm. **(c)** The assembly method of Janus solar dehumidification interface, and showed the hydrophilic properties of the upper surface. Water droplets spread rapidly on the upper surface of JSDI.

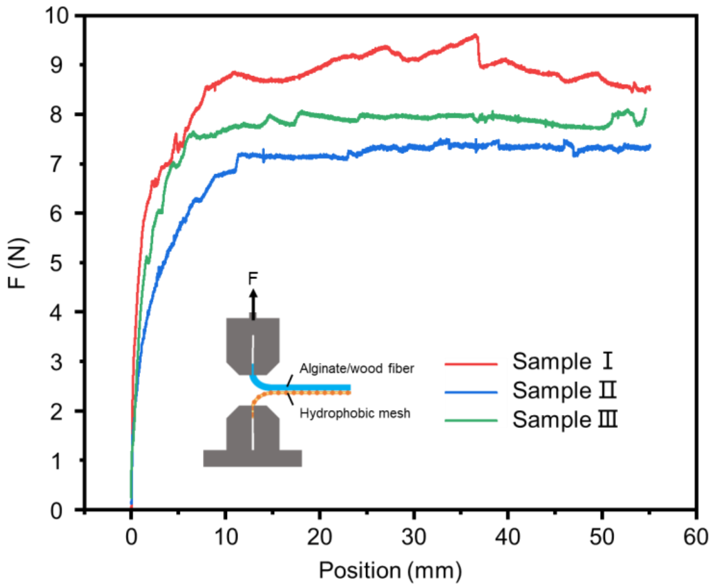


**Figure S2.** JSDI (with a width of 2 cm and a length of 6 cm) is placed on a an electromechanical universal testing machine to test the interfacial adhesion strength between the alginate/wood fiber layer and the hydrophobic mesh. The interfacial adhesion between the hydrophilic layer and the hydrophobic mesh was between 7-9 N.

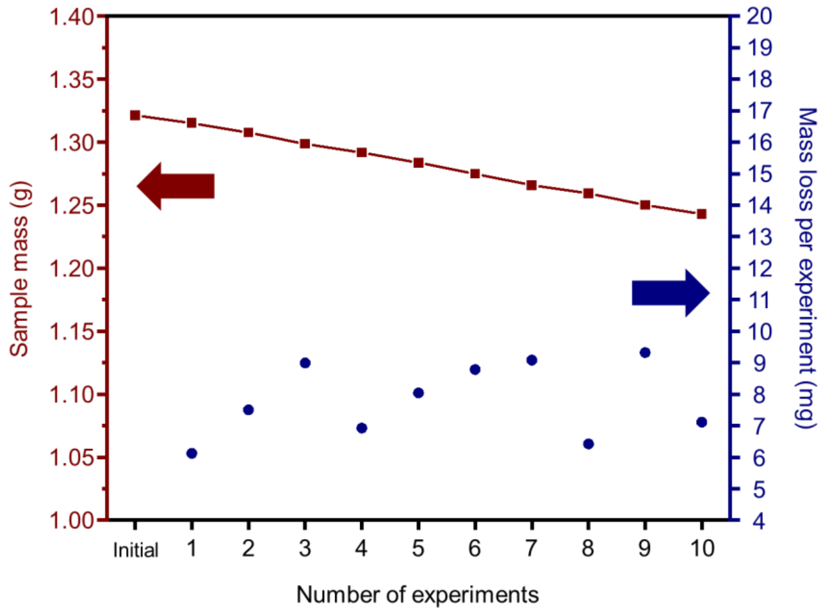


**Figure S3.** Take a hydrophilic layer with $CuCr_2O_4$ particles attached to the surface (sample size 5 cm × 5 cm) placed on a 5 ° slope, washed under water flow (flow rate of 10 mL/s) for 1 hour, then dried at 60℃ for 1 hour, and finally recorded the weight change. Repeat the above experiment 10 times. The percentage of total mass loss to the initial sample mass is 5.9%.

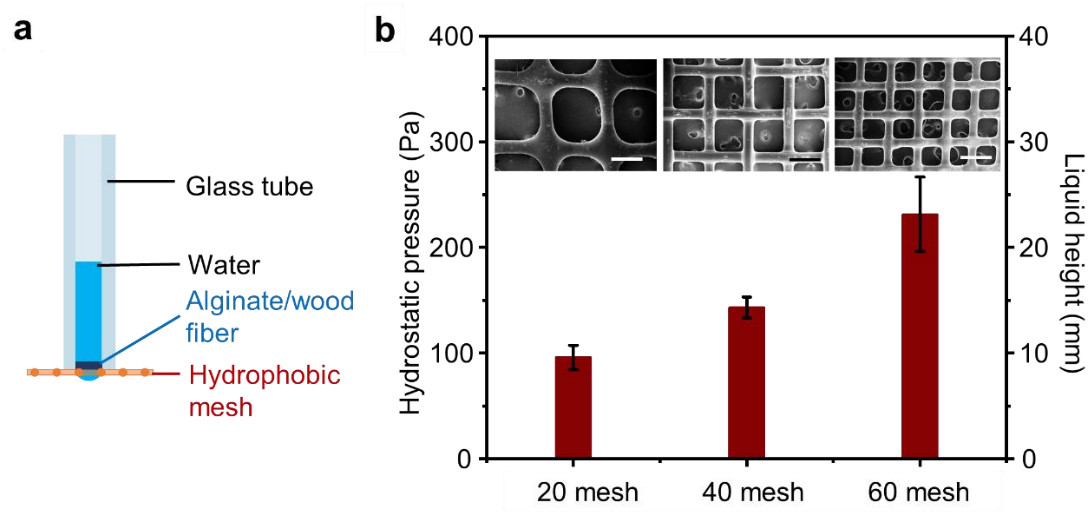


**Figure S4.** JSDI hydrostatic pressure measurement of water. **(a)** The hydrostatic pressure measurement experimental setup with glass tube, alginate/wood fiber and hydrophobic mesh. **(b)** Comparison of hydrostatic pressure of 20 mesh (96.04 ±11.43 Pa), 40 mesh (143.08 ± 9.99 Pa) and 60 mesh (231.28 ± 35.39 Pa). All of the hydrophobic meshes can support a liquid column exceeding 10 mm. The SEM images show the different the size of different mesh holes. The scale bar is 200 μm.

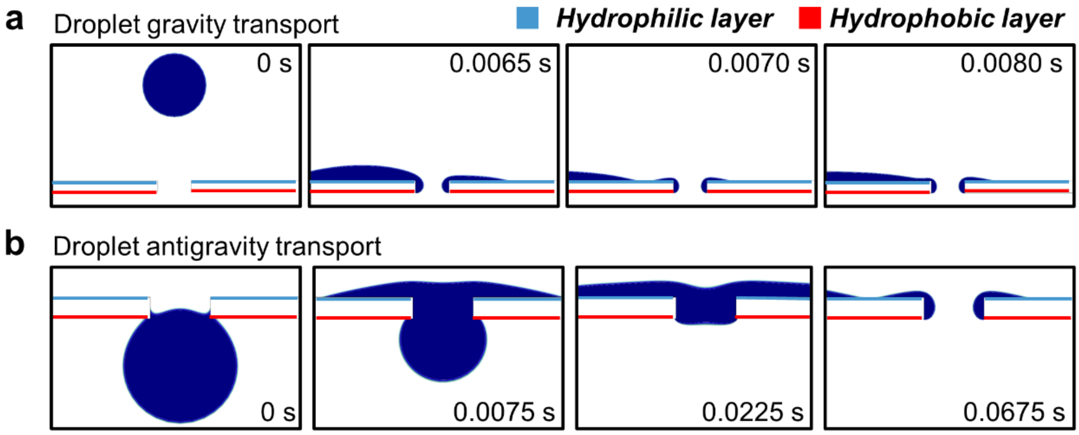


**Figure S5.** COMSOL Multiphysics for unidirectional liquid permeation is a simulation determined by the directionality of energy release from the surface. Gravitational **(a)** and inversed gravitational **(b)** transport of water droplets.

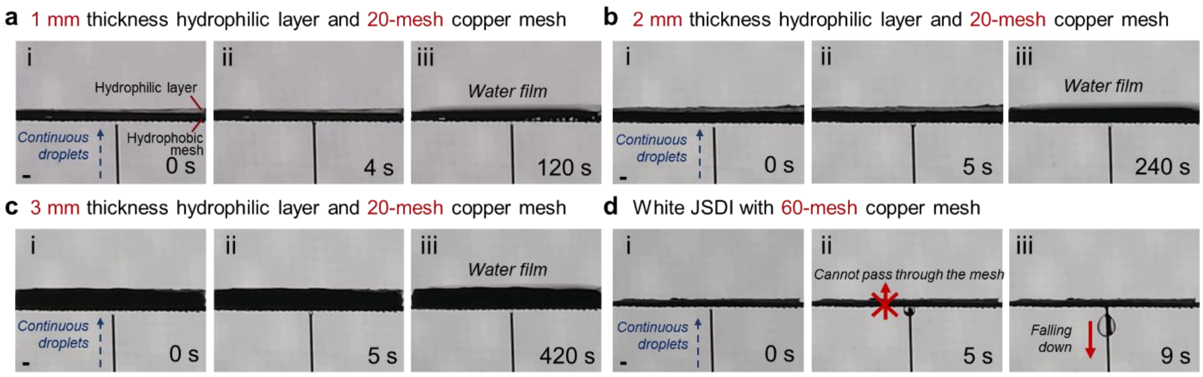


**Figure S6.** The continuous and anti-gravity droplets penetration through JSDI with different thickness of hydrophilic layer and mesh. The anti-gravity transport can be achieved for 1mm **(a)**, 2mm **(b)**, 3mm **(c)** of the hydrophilic layer. The 60-mesh copper mesh **(d)** is too small to cause the droplets to be blocked. The scale bar is 1 mm.

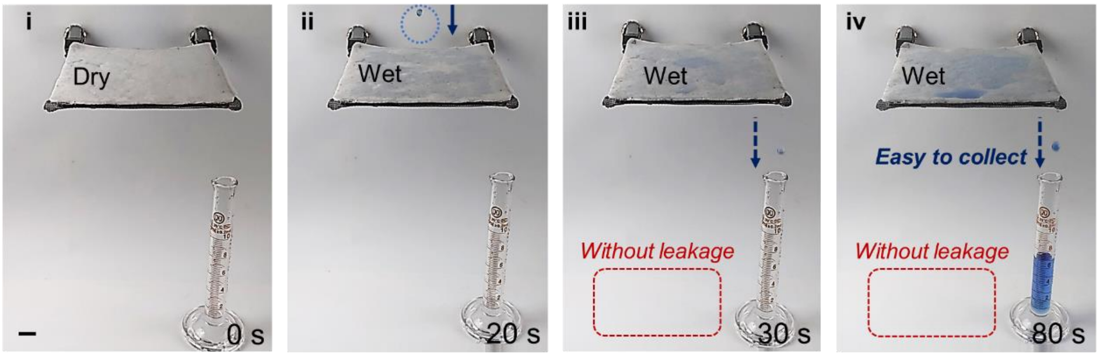


**Figure S7.** Under a dynamic water flow, the controllable water drainage via a siphoning structure on JSDI (Front View). The scale bar is 1 cm.

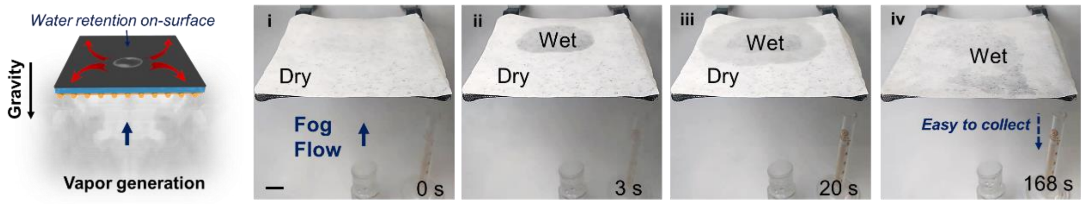


**Figure S8.** The anti-gravity microdroplet collecting process of JSDI (Front View). The scale bar is 1 cm.

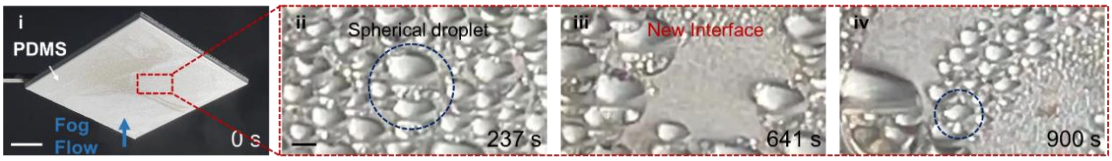


**Figure S9.** The PDMS coating is able to keep hydrophobic under 75°C steam. The scale bar of (i) is 1 cm, and that of (ii-iv) is 1 mm.

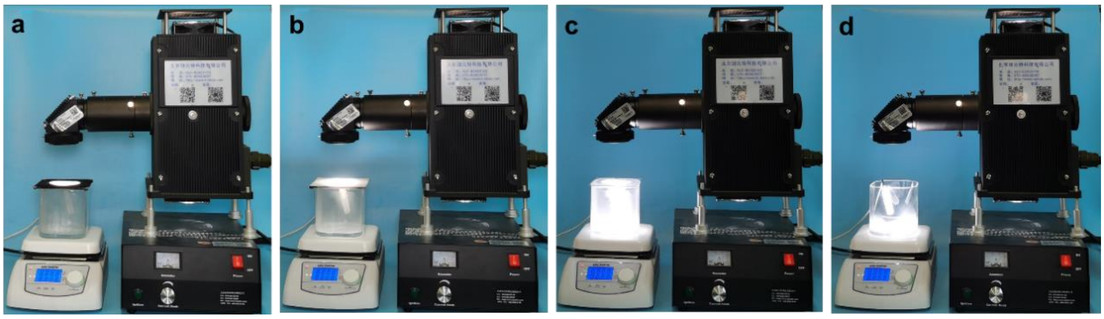


**Figure S10.** Photographs of the dehumidifying ability test of JSDI in laboratory. Four kinds of testing interface were selected as Black JSDI **(a)**, White JSDI **(b)**, closed system **(c)**, and open system **(d)**.

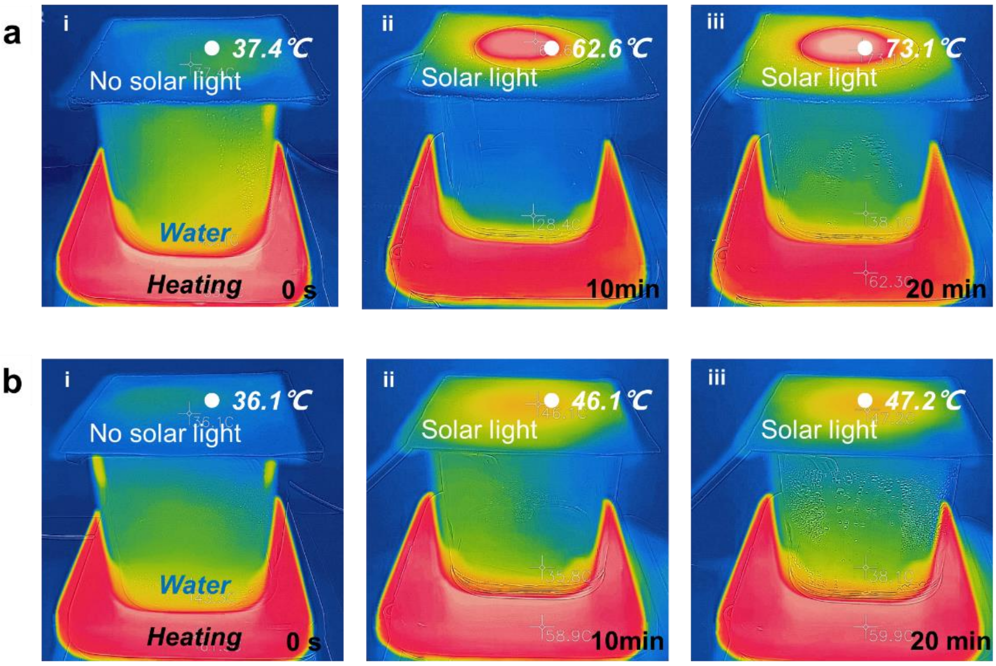


**Figure S11.** The increase process of the surface temperature of **(a)** Black JSDI and **(b)** White JSDI during the dehumidifying process was compared by using thermal images captured by a thermal infrared camera.

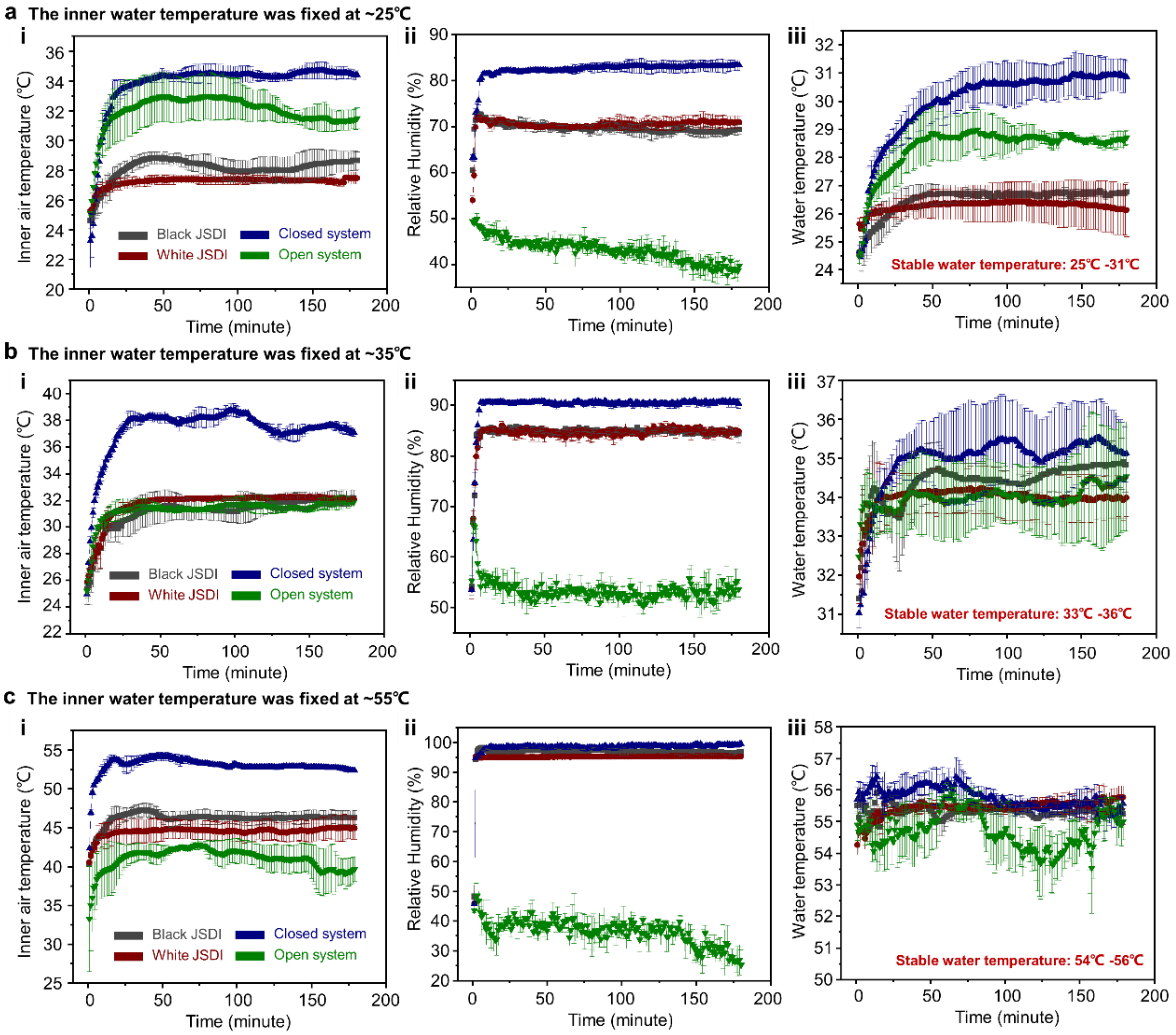


**Figure S12.** Comparison of dehumidification performance of the system corresponding to the four kinds of test interfaces (Black JSDI, White JSDI, closed system, open system), when the inner water temperature was fixed at **(a)** 25℃, **(b)** 35℃ and **(c)** 55℃. **(i)** The inner air temperature, **(ii)** the relative humidity and **(iii)** the water temperature are continuously recorded during the dehumidifying process.

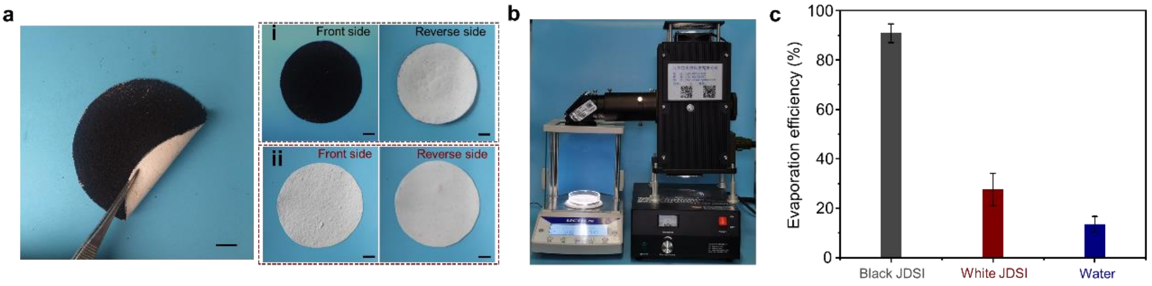


**Figure S13.** Compared the light-to-heat conversion efficiency of black and white JSDIs through an indoor experiment. **(a)** Alginate/wood fiber film with a diameter of 7 cm. **(i)** Black film with $CuCr_2O_4$ microparticle. **(ii)** White film without $CuCr_2O_4$ microparticle. The scale bar is 1 cm. **(b)** Photographs of the light-to-heat conversion efficiency experiment in laboratory. **(c)** Evaporation efficiencies of Black JDSI (90.8%), White JDSI (27.6%) and pure water (13.5%) under 1 sun light intensities.

**COMSOL simulation of vapor discharge of different container:**

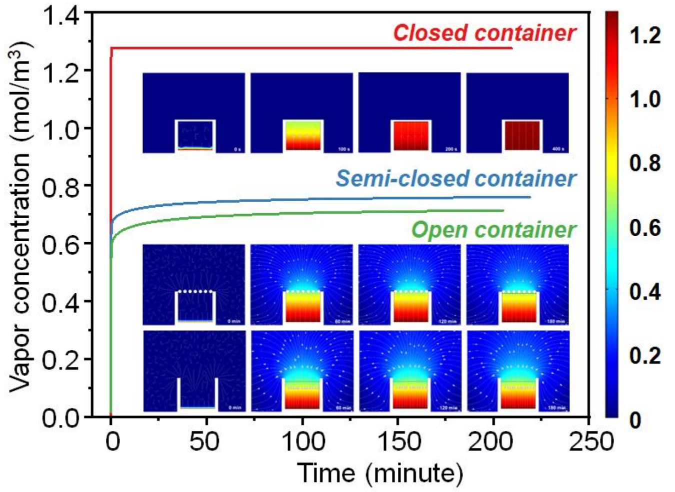


The COMSOL Multiphysics simulation is used to simulate the vapor discharge behavior of containers with different opening degrees. As shown in figure 4h, open, semi-closed, fully closed containers were considered in the simulation. Water evaporates from the bottom and the evaporative mass flux at the interface is given by diffusion of water vapor:

$$J_i = -D_i \nabla c_i$$

where $D$ is the diffusion constant of vapor in air and $c$ is the vapor concentration. Infinite element domain is used for specifying boundary conditions far away and the final vapor concentration is calculated. A simulated probe was used to monitor the vapor concentration at the outlet of the container. The simulation shows that the concentration of vapor at the outlet of the semi-open container is only about 9% higher than that of the fully open container, but it is about 45% lower than that of the fully closed container.

**Supplementary Movie:**

**Movie S1.** The continuous and anti-gravity droplets penetration through JSDI. (15×speed)

**Movie S2.** The continuous and anti-gravity droplets penetration through JSDI with different thickness of hydrophilic layer and mesh.

**Movie S3.** The unidirectional blocking of the upper fluids by JSDI. (10×speed)

**Movie S4.** The controllable water drainage via a siphoning structure on JSDI (Side View). (1.5×speed)

**Movie S5.** The controllable water drainage via a siphoning structure on JSDI (Front View). (5×speed)

**Movie S6.** The uncontrollable and undesired water penetration through a single-layer hydrophobic copper mesh (Front View). (5×speed)

**Movie S7.** The anti-gravity microdroplet collecting process of JSDI (Bottom View). (20×speed)

**Movie S8.** The anti-gravity microdroplet collecting process of JSDI (Front View). (2000×speed)

**Movie S9.** The microdroplet collecting process of single-layer hydrophobic copper mesh (Bottom View). (20×speed)

**Movie S10.** The direct comparison of the droplet pumping ability of JSDI and copper mesh (Side View). (10×speed)

**Movie S11.** The anti-gravity microdroplet collecting process of JSDI (Side View). (40×speed)

**Movie S12.** The COMSOL Multiphysics simulation of vapor transport within different systems.
